# Modulation of Adipogenesis and Oxidative Status by Quercetin and Ochratoxin A: Positive or Negative Impact on Rat Adipocyte Metabolism?

**DOI:** 10.3390/molecules24203726

**Published:** 2019-10-16

**Authors:** Viktoria Dobrocsyova, Katarina Krskova, Marcela Capcarova, Stefan Zorad

**Affiliations:** 1 Institute of Experimental Endocrinology, Biomedical Research Center, Slovak Academy of Sciences, Dúbravská Cesta 9, 845 05 Bratislava 4, Slovakia; ueenkrsk@savba.sk (K.K.); stefan.zorad@savba.sk (S.Z.); 2 Department of Animal Physiology, Faculty of Biotechnology and Food Sciences, Slovak University of Agriculture in Nitra, 949 76 Nitra, Slovakia; marcela.capcarova@uniag.sk

**Keywords:** adipose tissue, flavonoids, mycotoxins, reactive oxygen species, insulin sensitivity

## Abstract

(1) Background: Impaired adipose tissue function leads to the development of metabolic disorders. Reactive oxygen species play a key role in the regulation of adipogenesis and insulin-stimulated glucose uptake by adipocytes. Quercetin (QCT) regulates adipogenesis by affecting the redox state of preadipocytes. Ochratoxin A (OTA) is one of the most prevalent mycotoxins contaminating food. It has cytotoxic, genotoxic, pro-inflammatory, and anti-adipogenic effects. Antioxidants are believed to protect cells from the cytotoxicity and genotoxicity induced by OTA. The aim of this study was to investigate the effect of QCT and OTA application on preadipocyte differentiation, oxidative status, and adipocyte metabolism. (2) Methods: Primary rat preadipocytes were isolated from subcutaneous adipose tissue of Wistar rats. Gene expressions were determined by qPCR. Cell viability, reactive oxygen species (ROS) production, glucose uptake, and lipid accumulation were determined using commercially available kits. (3) Results: A dose-dependent inhibitory effect of QCT on adipogenic differentiation was observed, which was accompanied by a decrease in ROS production. Reduced ROS formation is closely related to impaired glucose uptake by adipocytes. (4) Conclusions: The results of this study indicate a key role of ROS in regulating adipogenesis and metabolic pathways, which is affected by the application of QCT and/or OTA.

## 1. Introduction

Adipose tissue is a dynamic endocrine organ that has the ability to influence the metabolism of the whole organism. It is involved in the regulation of energy homeostasis by storing excess metabolic substrates in the form of lipids, and releasing them at times of negative energy balance. Obesity contributes to the development of peripheral insulin resistance, which leads to impaired accumulation of excess metabolic substrates to hypertrophied adipocytes. Reactive oxygen species (ROS) have long been considered to be harmful byproducts, and their accumulation contributes to the development of degenerative diseases [1]. However, since the discovery of enzymes primarily involved in the formation of ROS (e.g., NADPH oxidases), the function of ROS has been reconsidered. At physiological concentrations, ROS act as signaling molecules [2]. The insulin-mimetic effects of ROS were discovered more than forty years ago [3,4]. Insulin itself stimulates the formation of ROS by activation of NADPH oxidase 4 (Nox4). Nox4 catalyzes the reduction of oxygen molecule to a superoxide radical [5]. In addition, Nox4 can also function as a “switch” between insulin-stimulated preadipocyte differentiation and proliferation [6]. During adipocyte differentiation, there is a parallel shift in redox equilibrium, which is reflected in increased ROS production, but also in increased activity of reducing agents. Mature adipocytes function at a higher level of redox equilibrium when compared to preadipocytes [7].

Quercetin (QCT) is one of the most commonly occurring bioactive flavonoids, found in various fruits, vegetables, and products of plant origin [8]. QCT has several pharmaceutical effects including cleavage of oxygen radicals, prevention of lipid peroxidation, and anti-adipogenic and anti-inflammatory effects [9,10]. QCT inhibits the differentiation of OP9 [11] and 3T3-L1 preadipocytes [12] into mature adipocytes by reducing lipid accumulation. In addition to this, it significantly reduces the expression of key transcription factors of adipogenesis, including adipocyte fatty acid-binding protein (aP2), peroxisome proliferator-activated receptor γ (PPARγ), and fatty acid synthase (FAS) [11,12]. The effect of QCT on adipogenic differentiation and adipocyte metabolism in vitro has been described mainly in the immortalized 3T3-L1 cell line. However, there is a lack of studies examining the adipogenic differentiation of primary rat cell cultures isolated from adipose tissue, which reflect adipose tissue physiology more faithfully than the immortalized cell line.

Ochratoxin A (OTA) is a mycotoxin produced by molds belonging to the genera *Aspergillus* (*Aspergillus ochraceus*, *Aspergillus carbonarius*) and *Penicillium* (*Penicillium verrucosum*). It is one of the most important mycotoxin contaminants in a wide variety of foodstuffs. OTA exerts nephrotoxicity, hepatotoxicity, teratogenicity, and immunosuppression [13]. Due to its widespread occurrence, the human population is constantly exposed to OTA. The presence of OTA has been demonstrated in various animal tissues, including human blood and breast milk [14]. It has been proven that OTA inhibits adipogenic differentiation of human mesenchymal stem cells (hMSCs) isolated from adipose tissue [15]. It has been proposed that antioxidants might protect cells from cytotoxicity and genotoxicity induced by OTA, however there is a lack of data regarding this hypothesis [16,17].

The aim of this study was to investigate the effect of QCT and OTA application on rat adipocyte metabolism, viability, and oxidative status, and to investigate potential regulatory mechanisms activated by administration of antioxidants and mycotoxins during preadipocyte differentiation.

## 2. Results

**Figure 1:**
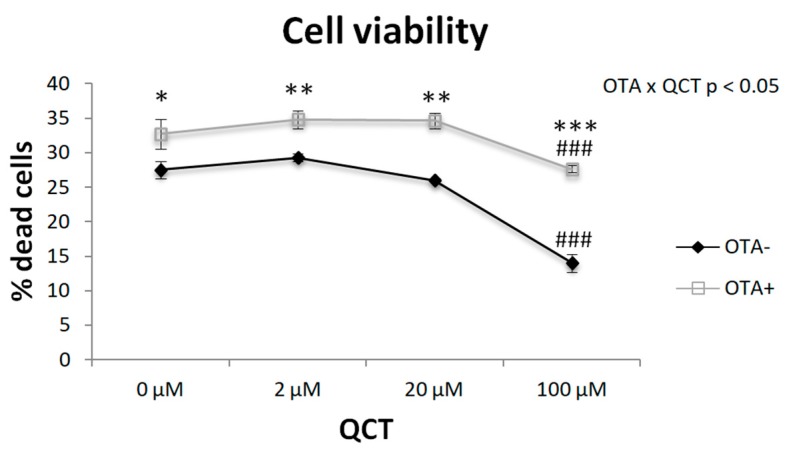
Effect of quercetin (QCT) at various concentrations with (OTA+) or without the addition of ochratoxin A (OTA-) in cell culture medium on the viability of differentiated primary rat preadipocytes. Cell viability was determined using a commercially available kit. The results, presented as mean ± mean error (SEM), were analyzed by two-way ANOVA with factors QCT and OTA, followed by Bonferroni post-hoc test. The level of statistical significance was considered for factor OTA * *p* < 0.05; ** *p* < 0.01; *** *p* < 0.001; and for factor QCT ### *p* < 0.001.

We measured cell viability after adding OTA to the cell culture medium in combination with different doses of QCT (Figure 1). Two-way ANOVA revealed a significant interaction of QCT and OTA application (*p* < 0.05). The presence of OTA increased the percentage of cell death by approximately 10–15%. Interestingly, the highest QCT concentration had a beneficial effect on cell survival in the control group (*p* < 0.001) as well as in the OTA-treated cell group (*p* < 0.001).

### 2.1. Adipogenesis

**Figure 2:**
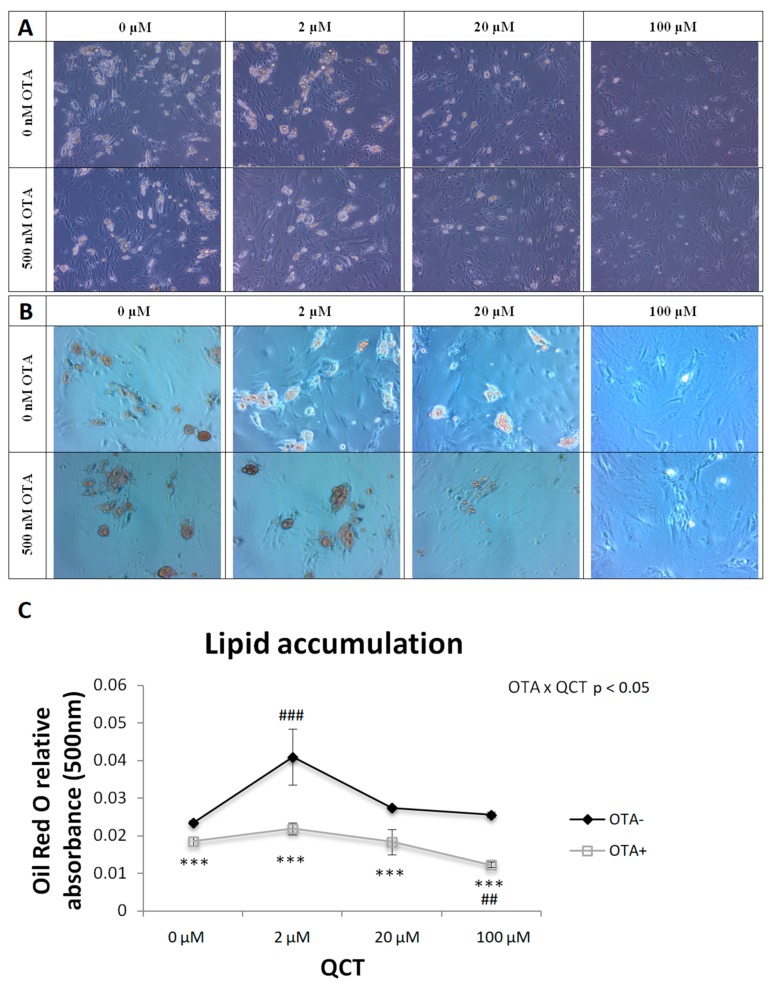
Histochemical staining of lipid accumulation by Oil Red O in differentiating primary rat preadipocytes cultured in medium with (OTA+) or without ochratoxin A (OTA-) and quercetin (QCT) at various concentrations. Cells were scanned both naive (**A**) and after histochemical staining with Oil Red O (**B**), magnification 100 ×. The absorbance of the Oil Red O eluate was measured at 500 nm (**C**). The results, presented as mean ± mean error (SEM), were analyzed by two-way ANOVA with factors QCT and OTA, followed by Bonferroni post-hoc test. The level of statistical significance considered for factor OTA was *** *p* < 0.001, and for factor QCT ## *p* < 0.01; ### *p* < 0.001.

The effect of QCT application at concentrations of 0, 2, 20, or 100 µM was observed on primary rat preadipocyte cells isolated from subcutaneous adipose tissue of Wistar rats with the addition of a vehicle or 500 nM OTA on adipogenic differentiation, expressed as lipid accumulation. Lipid accumulation was determined by histochemical staining with Oil Red O (Figure 2B). After elution of the dye from the cells, the absorbance of the eluate was measured at 500 nm and was evaluated statistically by a two-way analysis of variance (Figure 2C). A significant interaction of the two main factors, i.e., application of OTA and application of QCT (*p* < 0.05), was observed on lipid accumulation in primary rat preadipocytes. OTA suppressed adipogenic cell differentiation when compared to the control group (*p* < 0.001), with no significant effect of QCT at doses of 2 and 20 μM, whereas the highest QCT dose had an inhibitory effect on cell differentiation (*p* < 0.01). In contrast, adipogenesis was stimulated in the control group after addition of QCT at a concentration of 2 μM (*p* < 0.001), but higher doses of QCT had no significant effect on cell differentiation.

**Figure 3:**
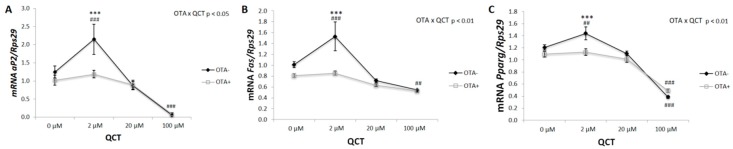
Gene expression of adipogenic markers in differentiated primary rat preadipocytes cultured with (OTA+) or without the addition of ochratoxin A (OTA-) in cell culture medium and with the addition of quercetin (QCT) at various concentrations. The expression of adipocyte Protein 2 (*aP2*; = fatty acid binding protein 4; **A**), fatty acid synthase (*Fas*; **B**) and peroxisome proliferator-activated receptor gamma (*Pparγ*; **C**) was determined by real-time PCR. Data were normalized to the gene expression of 40S ribosomal protein S29 (Rps29), the expression of which was not altered by the treatments. The results, presented as mean ± mean error (SEM), were analyzed by two-way ANOVA with factors QCT and OTA, followed by Bonferroni post-hoc test. The level of statistical significance considered for factor OTA was *** *p* < 0.001, and for factor QCT ## *p* < 0.01; ### *p* < 0.001.

The expression of the molecular markers of adipogenesis was in accordance with the results of Oil Red O staining (Figure 3). A significant interaction of the two main factors was detected on the mRNA levels of *aP2* (*p* < 0.05), *Fas* (*p* < 0.01), and *Pparγ* (*p* < 0.01). The expression of all three markers was increased after addition of 2 µM QCT to the control cell culture medium, while simultaneous addition of OTA prevented the induction of differentiation in this group. The highest concentration of QCT, 100 μM, had an inhibitory effect on adipogenic cell differentiation either with or without OTA. The gene expression of *aP2* (r = 0.687; *p* < 0.001), *Fas* (r = 0.772; *p* < 0.001) and *Pparγ* (r = 0.497; *p* < 0.001) positively correlated with the results of Oil Red O staining.

### 2.2. Adipokines

**Figure 4:**
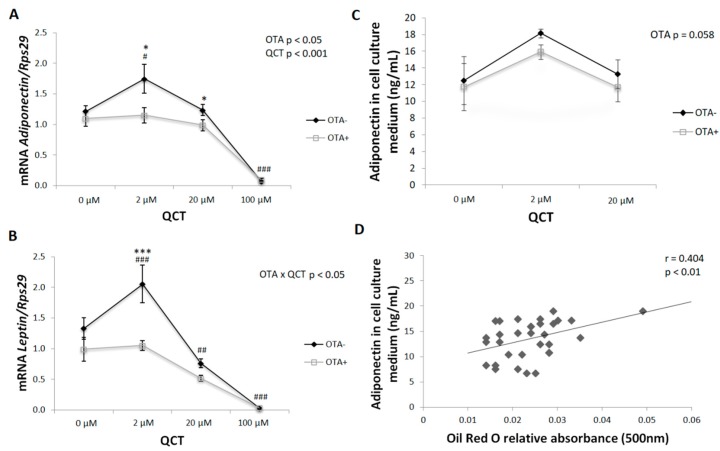
Gene expression of adipokines in differentiated primary rat preadipocytes cultured with (OTA+) or without the addition of ochratoxin A (OTA-) in cell culture medium and with the addition of quercetin (QCT) at various concentrations. The mRNA expression of *adiponectin* (**A**) and *leptin* (**B**) was determined by real-time PCR. Data were normalized to the gene expression of 40S ribosomal protein S29 (Rps29), the expression of which was not altered by the treatments. The amount of adiponectin released to the cell culture medium (**C**) was determined using a commercially available ELISA kit. The results, presented as mean ± mean error (SEM), were analyzed by two-way ANOVA with factors QCT and OTA, followed by Bonferroni post-hoc test. Correlation between cell culture adiponectin content and Oil Red O relative absorbance was analyzed using the Pearson correlation test (**D**). The level of statistical significance considered for factor OTA was * *p* < 0.05; *** *p* < 0.001, and for factor QCT # *p* < 0.05; ## *p* < 0.01; ### *p* < 0.001.

*Adiponectin* gene expression copied the changes observed in the expression of adipogenic markers in primary rat preadipocytes. A significant effect of both QCT (*p* < 0.05) and OTA (*p* < 0.001) application on adiponectin mRNA levels was detected. In the control group, adiponectin expression was stimulated by the addition of 2 µM QCT to the cell culture medium, which was prevented in the presence of OTA in the medium (Figure 4A). Similarly, the levels of adiponectin released into the medium, determined by ELISA, were in accordance with the mRNA expression. The inhibitory effect of OTA displayed borderline significance (*p* = 0.058) (Figure 4C). The gene expression (r = 0.568; *p* < 0.001) as well as adiponectin release to the culture medium positively correlated with the degree of cell differentiation evaluated by Oil Red O staining (Figure 4D).

The expression of *leptin*—another adipokine closely associated with the phenotype of mature adipocytes—reflected the previously observed changes. A significant interaction of the two main factors (*p* < 0.05) was detected. The presence of OTA in the medium prevented the stimulation of leptin gene expression after addition of 2 µM QCT. In contrast, higher doses of QCT had an inhibitory effect on leptin expression, regardless of whether OTA was also present in the medium (Figure 4B).

### 2.3. Glucose Uptake

**Figure 5:**
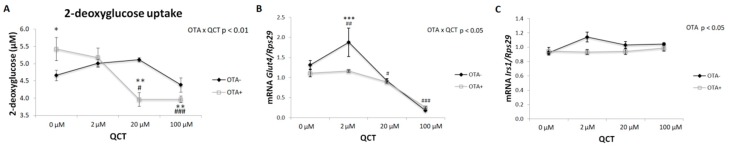
Insulin-stimulated glucose uptake by differentiated primary rat preadipocytes cultured with (OTA+) or without the addition of ochratoxin A (OTA-) in cell culture medium and with the addition of quercetin (QCT) at various concentrations (**A**). 2-deoxyglucose uptake was determined using a commercially available kit. The gene expression of glucose transporter 4 (*Glut4*; **B**) and insulin receptor substrate 1 (*Irs1*; **C**) was determined by real-time PCR. Data were normalized to the gene expression of 40S ribosomal protein S29 (*Rps29*), the expression of which was not altered by the treatments. The results, presented as mean ± mean error (SEM), were analyzed by two-way ANOVA with factors QCT and OTA, followed by Bonferroni post-hoc test. The level of statistical significance considered for factor OTA was * *p* < 0.05; ** *p* < 0.01; *** *p* < 0.001, and for factor QCT # *p* < 0.05; ## *p* < 0.01; ### *p* < 0.001.

Insulin-stimulated glucose uptake by differentiating cells was not affected by application of QCT at various doses in the absence of OTA in the medium (Figure 5A). However, after addition of 500 nM OTA to the culture medium, glucose uptake was significantly affected. Interestingly, in the control group, without the addition of QCT, glucose uptake was lower when compared to cells with the addition of OTA in the medium (*p* < 0.05) (Figure 5). In the presence of 2 µM QCT, glucose uptake was comparable in both groups. After QCT treatment at higher doses (20 and 100 μM), glucose uptake was significantly suppressed in the presence of OTA in the medium when compared to cells treated with vehicle only (*p* < 0.05; *p* < 0.001), but also when compared to cells treated with OTA but without the addition of QCT (*p* < 0.01).

Expression of glucose transporter 4 (*Glut4*) was influenced in a similar way to the expression of adipogenic markers, i.e., addition of 2 µM QCT to the control cells, stimulated the expression of *Glut4* (*p* < 0.01), which was prevented by co-treatment with OTA (Figure 5B). Higher doses of QCT had an inhibitory effect on *Glut4* expression both in the presence or absence of OTA in the culture medium. Insulin receptor substrate 1 (*Irs1*) expression was significantly lower after applying OTA to the culture medium when compared to the control group (*p* < 0.05). Addition of QCT had no significant effect on *Irs1* expression (Figure 5C).

### 2.4. ROS Production

**Figure 6:**
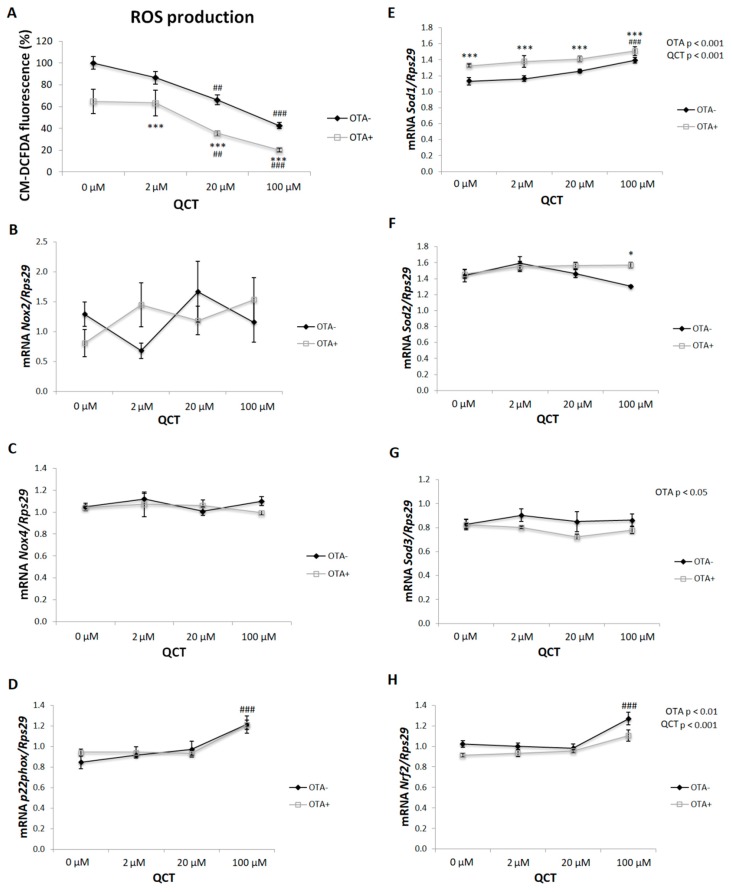
Production of reactive oxygen species (ROS) in differentiated primary rat preadipocytes cultured with (OTA+) or without the addition of ochratoxin A (OTA-) in cell culture medium and with the addition of quercetin (QCT) at various concentrations. The production of ROS was determined using a commercially available kit, and is expressed as a percentage of CM-H2DCFDA fluorescence relative to control (**A**). The expressions of NADPH oxidase 2 (*Nox2*; **B**), *Nox4* (**C**), p22 (**D**), superoxide dismutase 1 (*Sod1*; **E**), *Sod2* (**F**), *Sod3* (**G**) and nuclear factor erythroid 2–related factor 2 (*Nrf2*; **H**) were determined by real-time PCR. Data were normalized to the gene expression of 40S ribosomal protein S29 (Rps29), the expression of which was not altered by the treatments. The results, presented as mean ± mean error (SEM), were analyzed by two-way ANOVA with factors QCT and OTA, followed by Bonferroni post-hoc test. The level of statistical significance considered for factor OTA was * *p* < 0.05; *** *p* < 0.001, and for factor QCT ## *p* < 0.01; ### *p* < 0.001.

ROS production, expressed as a percentage of fluorescence of the oxidized impermeable molecular probe CM-H_2_DCF, was significantly affected by the addition of OTA (*p* < 0.001) and QCT (*p* < 0.001) to the cell culture medium (Figure 6A). The presence of OTA reduced the formation of ROS when compared to the control cells (*p* < 0.001). A low dose of QCT (2 μM) had no effect on ROS production in either group, while higher doses of QCT (20 and 100 μM) reduced ROS production in a dose-dependent manner (*p* < 0.01; *p* < 0.001). The expressions of both pro- and antioxidant markers were also determined. The expressions of *Nox2* and *Nox4* were not significantly affected by the administration of QCT, and/or OTA (Figure 6C,E). Gene expression of *p22* (an essential component of NADPH oxidase) was only affected by the addition of the highest dose of QCT to the cell medium (*p* < 0.001) (Figure 6G). A significant stimulatory effect of OTA treatment (*p* < 0.001) on superoxide dismutase 1 (*Sod1*) expression was detected (Figure 6B). The highest QCT concentration increased *Sod1* mRNA levels (*p* < 0.001). *Sod2* expression was increased in cells cultured in the presence of OTA with the addition of 100 µM QCT to the medium (*p* < 0.05) (Figure 6D). OTA treatment had an inhibitory effect on *Sod3* expression when compared to control cells (*p* < 0.05) (Figure 6F). The expression of nuclear factor erythroid 2–related factor 2 (*Nrf2*) was reduced when 500 nM OTA was added to the medium (*p* < 0.01). In the control group of cells, *Nrf2* expression was increased by the addition of 100 μM QCT to the cell culture medium (*p* < 0.001) (Figure 6H).

## 3. Discussion

It has been shown that OTA induces cytotoxicity, genotoxicity, and reactive oxygen species in kidney cells [18]. Therefore, cell viability was measured after adding OTA to the culture medium in combination with different doses of QCT. A slight cytotoxic effect of 500 nM OTA in rat primary adipocyte culture was confirmed in this study. Surprisingly, the highest concentration of QCT showed a protective effect on cell survival.

OTA inhibits adipogenic differentiation of hMSCs isolated from adipose tissue by activation of the extracellular-signal-related kinase, resulting in the inhibition of *PPARγ* and subsequent down-regulation of adipogenesis [15]. Furthermore, OTA reduces the expression of adipocyte-specific markers in hMSCs [15]. In this study, the inhibitory effect of OTA on lipid accumulation and expression of adipogenic markers were confirmed in primary rat preadipocytes isolated from subcutaneous adipose tissue. Concurrent application of QCT could not reverse this process. An interesting finding was the dose-dependent effect of QCT on lipid accumulation and expression of adipogenic markers in the control group. While the lowest 2 μM dose of QCT stimulated adipogenic differentiation, higher doses of QCT displayed an inhibitory effect on these parameters. This is in accordance with other studies, where low doses of QCT induced lipid accumulation and increased the expression of adipogenic markers, e.g., *aP2* in hMSCs [19,20]. On the other hand, studies that examined adipogenic differentiation on the immortalized 3T3-L1 cell line showed an exclusively inhibitory effect of QCT [21,22]. Comparison of these studies points out the differences in the adipogenic differentiation of the widely used 3T3-L1 mouse cell line compared to the primary preadipocytes isolated from rat adipose tissue.

During terminal differentiation of adipocytes, there is an increase in the expression of genes characteristic for the phenotype of mature adipocytes. Mature adipocytes are able to produce adipokines [23]. Accordingly, expression of adipokines (*adiponectin* and *leptin*) was stimulated after application of QCT at a 2 μM dose and inhibited in the presence of 100 μM QCT, which positively correlated with lipid accumulation in cells. In the process of differentiation, ROS are key signaling molecules. Elimination of ROS formation results in disruption of adipogenic differentiation [24,25]. The dose-dependent effect of QCT on inhibition of ROS production under basal conditions was confirmed in this study, as well as in cells with OTA added to the culture medium. However, the expected increase in ROS formation after OTA application was not confirmed in this study. Paradoxically, the presence of OTA in the medium inhibited the formation of ROS when compared to basal conditions. On the other hand, reduced ROS formation, either due to the addition of 20 and 100 μM QCT, and/or the concomitant addition of 500 nM OTA to the culture medium, may have contributed to the reduced adipogenic differentiation of primary rat preadipocytes. Mature adipocytes function at a higher level of redox equilibrium when compared to preadipocytes [7]. The expressions of both pro- and antioxidant markers were not changed by OTA, with the exception of *Sod1*. A significant increase in *Sod1* gene expression after addition of OTA was observed when compared to basal conditions, which may have at least partially contributed to the decreased ROS formation observed in this experimental group. To the best of our knowledge, the effect of OTA application on ROS production either in adipocytes or adipose tissue has not been evaluated yet. This study was the first to describe reduced ROS production by differentiated primary rat preadipocytes upon 500 nM OTA treatment. The results of this study are in contrast with other studies that evaluated the prooxidative effect of OTA application in vitro on many cell types [26], including monkey kidney cells (Vero E6 cell line) [18], peripheral blood mononuclear cells from human (hPBMC) [17], GES-1 cells derived from a human fetal gastric mucosa epithelium [27], and HepG2 cells [28]. On the other hand, in these studies, a significantly higher concentration of OTA was applied within the micromolar range for a shorter period of time, from 24 h up to 72 h. In this study, 500 nM OTA was applied based on the study of Lim et al. [15], since in that study they showed an inhibitory effect of OTA (at 500 nM) on adipocyte differentiation without affecting cellular toxicity. Furthermore, in this study, the cells were exposed to OTA for a much longer period of time (13 days) in comparison to the aforementioned studies (24–72 h). Hence, this study may be considered as an evaluation of chronic exposure to OTA at lower doses, where other mechanisms may have been involved in short-term studies that applied high doses of OTA.

Several lines of evidence suggest an insulin-mimetic effect of ROS in insulin-sensitive tissues. In addition, upon insulin stimulation, *Nox4* is activated simultaneously with the insulin signaling cascade, resulting in a subsequent formation of ROS, which facilitates signal transduction [29,30,31]. Since a change in the redox equilibrium of the differentiating preadipocytes was detected, insulin-stimulated glucose uptake and the genes involved in insulin signaling were also measured. Surprisingly, cells cultured in the presence of OTA showed an improved insulin-stimulated glucose uptake when compared to cells cultured under basal conditions. Regarding the effect of QCT, there was no significant effect on glucose uptake by adipocytes under basal conditions. In contrast, cells cultured in medium with the addition of 500 nM OTA showed a dose-dependent response to the effect of QCT. Glucose uptake was stimulated at zero and low dose of QCT (2 μM), while at higher doses (20 and 100 μM), an inhibitory effect on insulin-stimulated glucose uptake by the cells was observed. Accordingly, the formation of ROS was significantly suppressed in these groups, which indicates its key role in the insulin-stimulated glucose uptake in adipocytes. To the best of our knowledge, this has been the first study to describe the stimulatory effect of 500 nM OTA on insulin-stimulated glucose uptake by adipocytes. The mechanisms underlying this observation need further investigation.

## 4. Conclusions

In this study, a dose-dependent action of QCT on adipogenic cell differentiation was observed, having a stimulatory effect at low doses and inhibitory action at high doses. This observation is consistent with studies on hMSCs, but is in contrast with studies on 3T3-L1 mouse cells showing an exclusively inhibitory effect of QCT on adipogenic differentiation. These results indicate a different response to QCT application during adipogenic differentiation of the immortalized mouse 3T3-L1 cell line when compared to primary rat preadipocytes, which in this respect are physiologically more similar to hMSCs. Furthermore, the inhibitory effect of OTA on adipogenic differentiation of primary rat preadipocytes was also confirmed, and could not be restored by concomitant QCT treatment. The expression of adipokines copied the expression of adipogenic markers. The results of this study point out the key role of ROS not only in adipogenic differentiation of precursor cells, but also in insulin-stimulated glucose uptake by cells, which is an essential function of mature adipocytes. Both QCT and OTA caused a shift in the redox balance of adipocytes, which might impair cell function depending on the dose of drug.

## 5. Materials and Methods

### 5.1. Isolation of Primary Rat Preadipocytes from Subcutaneous Adipose Tissue of Wistar Rats

Subcutaneous adipose tissue samples were collected from Wistar rats and placed in sterile phosphate buffered saline (PBS) pH 7.4 (Gibco, Thermo Fischer Scientific, Rockford, IL, USA). Samples were cut with sterile scissors and placed in sterile buffer 25 mM NaHCO_3_, 12 mM KH_2_PO_4_, 1.4 mM CaCl_2_ · 2H_2_O (Lachema, Brno, Czech Republic), 1.2 mM MgSO_4_ (Mikrochem, Pezinok, Slovakia), 4.8 mM KCl (Slavus, Podunajske Biskupice, Slovakia), 120 mM NaCl (Serva, Heidelberg, Germany), 5 mM glucose, 2.5% bovine serum albumin (Sigma-Aldrich, St. Louis, USA), 100 U/mL penicillin, 100 µg/mL streptomycin, 0.25 µg/mL amphotericin B (Gibco, Thermo Fischer Scientific, Rockford, IL, USA), and 1 mg/mL collagenase (Gibco, Thermo Fischer Scientific, Rockford, IL, USA) and pH adjusted to 7.4. The samples were incubated for 60 min in a 37 °C water bath. Subsequently, 20 mL of DMEM 4.5 g/L D-glucose (Dulbecco’s Modified Eagle Medium; Gibco, Thermo Fischer Scientific, Rockford, IL, USA) supplemented with 10% fetal bovine serum (FBS; Gibco, Thermo Fischer Scientific, Rockford, IL, USA), 100 U/mL penicillin, 100 µg/mL streptomycin, and 0.25 µg/mL amphotericin B (Gibco, Thermo Fischer Scientific, Rockford, IL, USA) was added. The samples were filtered through sterile gauze and centrifuged for 10 min at 1000 rpm at room temperature. The pellet was suspended in 10 mL culture medium and re-filtered through a sterile nylon filter with pore size 30 µm (MACS^®^, Miltenyi Biotec GmbH, Bergisch Gladbach, Germany) and centrifuged for 5 min at 1000 rpm at room temperature. The pellet was suspended in 5 mL of sterile erythrocyte lysis buffer: 154 mM NH_4_Cl (Slavus, Podunajske Biskupice, Slovakia), 10 mM KHCO_3_ (Lachema, Brno, Czech Republic), EDTA (Sigma-Aldrich, St. Louis, MO, USA), 100 U/mL penicillin, 100 µg/mL streptomycin, and 0.25 µg/mL amphotericin B (Gibco, Thermo Fischer Scientific, Rockford, IL, USA). After 5 min of incubation, the samples were centrifuged for 5 min at 1000 rpm at room temperature. The pellet was suspended in the culture medium.

### 5.2. Adipogenic Differentiation Protocol

**Figure 7:**
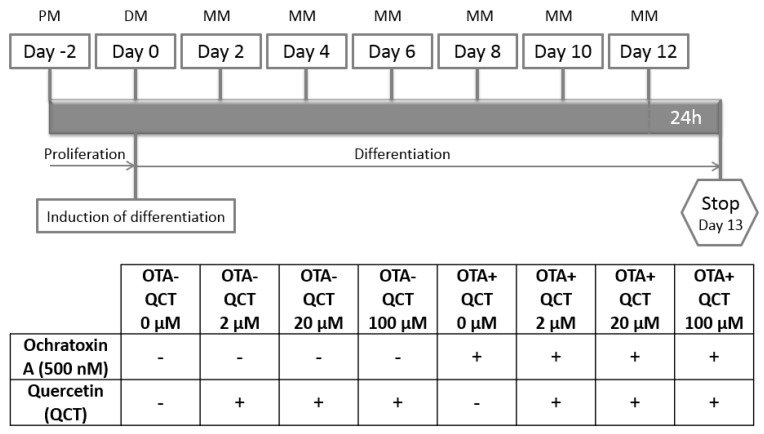
General scheme of research methodology. PM—proliferation medium, DM—differentiation medium, MM—maintenance medium.

Primary rat preadipocytes were cultured in proliferation medium consisting of Gibco™ DMEM 4.5 g/L D-glucose supplemented with 10% FBS, 100 U/mL penicillin, 100 µg/mL streptomycin and 0.25 µg/mL amphotericin B. Cells were cultured in culture flasks in an incubator at 37 °C in an atmosphere with 5% CO_2_ concentration. Cells were seeded into 75 cm^2^ culture flasks (TPP, Trasadingen, Switzerland) at a density of 5000 cells/cm^2^. After 24 h, the culture medium was replaced with fresh medium in order to remove any dead cells and rinse out the residue of cell freezing medium (Sigma-Aldrich, St. Louis, MO, USA) residues from the cell culture. Subsequently, the medium was changed every 48 h. After reaching ~80% confluency, cells were passaged and plated on 12 well plates (Greiner CELLSTAR^®^, Sigma-Aldrich, St. Louis, MO, USA) at a density of 3500 cells/cm^2^. Two days postconfluently, adipogenic cell differentiation was induced by changing the proliferation medium to differentiation medium, which consisted of Gibco™ DMEM containing 4.5 g/L D-glucose supplemented with 10% FBS, 100 U/mL penicillin, 100 µg/mL streptomycin, 0.25 µg/mL amphotericin B, 10 µg/mL insulin, 1 µM dexamethasone, 0.5 mM 3-isobutyl-1-methylxanthine (IBMX), and 2 µM rosiglitazone (Sigma-Aldrich, St. Louis, MO, USA). Cells were maintained in maintenance medium which consisted of Gibco™ DMEM containing 4.5 g/L D-glucose supplemented with 10% FBS, 100 U/mL penicillin, 100 µg/mL streptomycin, 0.25 µg/mL amphotericin B, 10 µg/mL insulin, and 1 µM dexamethasone. Subsequently, 500 nM Ochratoxin A (OTA; Sigma-Aldrich, St. Louis, MO, USA; dissolved in ethanol and PBS 1:1) and quercetin (QCT; Sigma-Aldrich, St. Louis, MO, USA; dissolved in DMSO and PBS 1:4) at concentrations 0, 2, 20, and 100 µM were added to the culture medium every 48 h after induction of differentiation throughout the experiment (Figure 7).

Cell cultures were divided into the following groups:

Cells were cultured in an incubator at 37 °C in 12 well plates at a 5% concentration of CO_2_ for 16 days before they were used immediately for ROS measurement, determination of cell viability, determination of insulin-stimulated glucose uptake by adipocytes, stained with Oil Red O, or frozen and stored at −80 °C until further use.

### 5.3. RNA Isolation and Real-Time PCR

Total RNA was isolated from differentiated primary rat adipocytes using RNeasy Universal Plus Mini Kit (Qiagen, Valencia, CA, USA) and reverse transcription was performed using Maxima First Strand cDNA Synthesis Kit (Thermo Fisher, Waltham, MA, USA) according to the manufacturer’s protocol. Real-time PCR was carried out by applying Maxima Sybr Green qPCR Master Mix (Thermo Fisher, Waltham, MA, USA) and run on an ABI 7900HT thermal cycler (Applied Biosystems, Life Technologies, Carlsbad, CA, USA) using rat-specific primer pairs, as shown in Table 1. Data were normalized to the expression of housekeeping gene ribosomal protein S29 (*Rps29*) which was not altered by QCT or OTA.

### 5.4. ELISA

Adiponectin production was measured in the cell culture medium using a commercially available Rat Adiponectin ELISA Kit (ab108784, Abcam, Cambridge, UK) according to the manufacturer’s instructions. The absorbance at a wavelength of 450 nm was measured immediately on a microplate reader SynergyTM H4 Hybrid Reader fluorometer (Biotek, Winooski, VT, USA).

### 5.5. ROS Production

Reactive oxygen species were determined using a commercially available CM-H_2_DCFDA General Oxidative Stress Indicator kit (C6827, Thermo Fisher Scientific, Rockford, IL, USA) according to the manufacturer’s instructions. CM-H_2_DCFDA passively diffused into cells, where its hydrolyzation and oxidation yielded a fluorescent signal that was measured at 485 nm (excitation) and 535 nm (emission) using a SynergyTM H4 Hybrid Reader fluorometer (Biotek, Winooski, VT, USA).

### 5.6. Cell Viability

Cell viability was determined using a commercially available LIVE/DEAD™ Viability/Cytotoxicity Kit for mammalian cells (L3224, Thermo Fisher Scientific, Rockford, IL, USA) according to the manufacturer’s instructions. The fluorescence of ethidium homodimer (EthD-1) was measured at (ex/em ~495 nm/~635 nm) using a SynergyTM H4 Hybrid Reader fluorometer (Biotek, Winooski, VT, USA).

### 5.7. Insulin-Stimulated Glucose Uptake

Glucose uptake by fat cells was determined using a commercially available Glucose Uptake Assay Kit (Colorimetric) (Abcam, Cambridge, UK) according to the manufacturer’s instructions. Measurement of OD_412nm_ was carried out using SynergyTM H4 Hybrid Reader (Biotek, Winooski, VT, USA). Glucose uptake is expressed as the concentration of 2-deoxyglucose in µM in cells.

### 5.8. Oil Red O Staining

Adipogenic differentiation expressed as fat accumulation in primary rats preadipocytes was determined using a commercially available Oil Red O Stain Kit (Lipid Stain) (Abcam, Cambridge, UK) according to the manufacturer’s instructions. Oil Red O was eluted from the cells with 100% isopropanol for 10 min, and the absorbance of the eluate was measured at 500 nm using a SynergyTM H4 Hybrid Reader (Biotek, Winooski, VT, USA).

### 5.9. Statistical Analysis

The results are presented as mean ± SEM. Analysis of normally distributed data was performed using Shapiro–Wilk test. Differences between experimental groups were analyzed by two-way ANOVA with factors QCT and OTA using the software SigmaStat 3.5 (Systat Software, San Jose, CA, USA). Whenever interaction between the main factors (QCT and OTA) reached significance, a Bonferroni post-hoc test was applied. Non-normally distributed data were subjected to natural logarithm transformation prior to statistical analysis. Correlations between variables were analyzed using the Pearson correlation test. Overall level of statistical significance was reached at * *p* < 0.05; ** *p* < 0.01; *** *p* < 0.001.

## Figures and Tables

**Table 1:** Primers used in qPCR.

Gene		Primer Sequences
***Adiponectin***	Fw	5′-ACC CTT GGC AGG AAA GGA-3′
	Rv	5′-CCT ACG CTG AAT GCT GAG TGA T-3′
***aP2*** ***(FABP4)***	Fw	5′-AGC GTA GAA GGG GAC TTG GT-3′
	Rv	5′-ATG GTG GTC GAC TTT CCA TC-3′
***Fas***	Fw	5′-GAG TCT GTC TCC CGC TTG AC-3′
	Rv	5′-TGG AAA TGA GGG CCA TAG TC-3′
***Glut4***	Fw	5′-TTT CCA GTA TGT TGC GGA TG-3′
	Rv	5’-TCA GTC ATT CTC ATC TGG CC-3’
***Irs-1***	Fw	5′-CCA AGG GCT TAG GTC AGA CAA A-3′
	Rv	5′-GCC TCA GAG TTG AGC TTC ACA A-3′
***Leptin***	Fw	5′-TCC AGG ATG ACA CCA AAA CC-3′
	Rv	5′-GAA GGC AAG CTG GTG AGG AT-3′
***Nox2***	Fw	5′-TGA TCA TCA CAT CCT CCA CCA A-3′
	Rv	5′-GAT GGC AAG GCC GAT GAA-3′
***Nox4***	Fw	5′-CTG CAT CTG TCC TGA ACC TCA A-3′
	Rv	5′-TCT CCT GCT AGG GAC CTT CTG T-3′
***Nrf2***	Fw	5′-GTT GAG AGC TCA GTC TTC AC-3′
	Rv	5′-CAG AGA GCT ATC GAG TGA CT-3′
***p22***	Fw	5′-TGG CCT GAT CCT CAT CAC AG-3′
	Rv	5′-AGG CAC GGA CAG CAG TAA GT-3′
***Pparγ***	Fw	5′-AGG ATT CAT GAC CAG GGA GTT-3′
	Rv	5′-AGC AAA CTC AAA CTT AGG CTC CAT-3′
***Rps29***	Fw	5′-GCT GAA CAT GTG CCG ACA CT-3′
	Rv	5′-GGT CGC TTA GTC CAA CTT AAT GAA-3′
***Sod1***	Fw	5′-CAC TCT AAG AAA CAT GGC G-3′
	Rv	5′-CTG AGA GTG AGA TCA CAC G-3′
***Sod2***	Fw	5′-TTC AGC CTG CAC TGA AG-3′
	Rv	5′-GTC ACG CTT GAT AGC CTC-3′
***Sod3***	Fw	5′-CTT GAC CTG GTT GAG AAG ATA G-3′
	Rv	5′-GAT CTG TGG CTG ATC GG-3′
